# ASO Author Reflections: Von Willebrand Factor for Assessment of Portal Hypertension and Perioperative Risk in Resectable HCC

**DOI:** 10.1245/s10434-024-15781-0

**Published:** 2024-07-16

**Authors:** D. Pereyra, M. Mandorfer, P. Starlinger

**Affiliations:** 1 Division of Visceral Surgery, Department of General Surgery, Medical University of Vienna, General Hospital, Vienna, Austria; 2 Division of Gastroenterology and Hepatology, Department of Medicine III, Medical University of Vienna, General Hospital, Vienna, Austria; 3 Vienna Hepatic Hemodynamic Lab, Medical University of Vienna, General Hospital, Vienna, Austria; 4 Division of Hepatobiliary and Pancreatic Surgery, Department of Surgery, Mayo Clinic, Rochester, MN USA

## Past

Risk stratification for posthepatectomy liver failure (PHLF) is essential for optimizing outcome in patients undergoing liver resection. This is particularly important in patients treated for hepatocellular carcinoma (HCC) owing to their increased risk for postoperative complications from underlying advanced chronic liver disease (ACLD). The estimation of functional liver reserve is often imprecise owing to ACLD and portal hypertension, which represent significant challenges in preoperative risk assessment.^1^ Consequently, noninvasive but resource-intensive tests, such as indocyanine green clearance evaluation, along with minimally invasive hepatic venous pressure gradient measurement, which requires considerable expertise, remain essential components in the preoperative workup in this high-risk group.

## Present

This retrospective analysis^2^ aimed to optimize preoperative risk assessment in HCC patients, using Von Willebrand factor antigen (vWF-Ag), a broadly available noninvasive biomarker for PHLF. vWF-Ag has previously been evaluated and validated for predicting PHLF in cohorts of all-comers for liver resection and displayed high diagnostic accuracy for clinically significant portal hypertension (CSPH) in patients with compensated ACLD.^3,4^ The present data facilitated the establishment of cutoffs for noninvasive CSPH assessment as well as PHLF risk stratification in patients with HCC. Moreover, associations of vWF-Ag with early disease recurrence and short overall survival in HCC patients were observed.

In the present study, which includes a large cohort of compensated and potentially resectable HCC patients, the applied cutoffs for vWF-Ag identified risk groups of particular clinical interest. A low-risk cutoff (vWF-Ag ≤ 182%) ruled out CSPH and showed a negative predictive value of 100% for PHLF. Thus, it identifies a subgroup of patients for whom additional resource-intensive preoperative evaluation can be safely omitted. In contrast, vWF-Ag levels of > 291% were indicative of CSPH; 57% of patients within this highest-risk group developed PHLF, and 50% of these patients did not survive the first postoperative year. The positive likelihood ratio for preoperative vWF-Ag > 291% of 6.05 indicates a substantial increase in the probability for PHLF from 20% (pre-test) to 60% (post-test). Importantly, the findings on PHLF risk were validated in an external cohort of HCC patients undergoing liver resection.

## Future

The observations made in these well-characterized sets of HCC patients are highly relevant for clinical decision-making. As patient selection based on the individual risk for PHLF and disease recurrence is crucial for improving outcomes of HCC patients considered for liver resection, including vWF-Ag in the process of preoperative risk evaluation adds granularity to the current assessments. Notably, vWF-Ag helps define a low-risk cohort in which HVPG testing may not be necessary as the risk of CSPH is minimal and outcomes are excellent. Conversely, patients in the highest-risk group are unlikely to benefit from liver resection and may be better served by alternative treatment options, though the optimal plan of action requires further evaluation.^5^ In summary, use of vWF-Ag testing might ultimately allow for more individualized treatment of HCC patients, while also being a low-cost, noninvasive, and readily available biomarker.
